# Molecular Mechanisms of Chemotherapy Resistance in Head and Neck Cancers

**DOI:** 10.3389/fonc.2021.640392

**Published:** 2021-05-07

**Authors:** Yuzuka Kanno, Chang-Yu Chen, Hsin-Lun Lee, Jeng-Fong Chiou, Yin-Ju Chen

**Affiliations:** ^1^ Division of Molecular Regulation of Inflammatory and Immune Disease, Research Institute for Biomedical Sciences, Tokyo University of Science, Chiba, Japan; ^2^ Department of Medicinal and Life Sciences, Faculty of Pharmaceutical Sciences, Tokyo University of Science, Chiba, Japan; ^3^ Graduate School of Medicine, The University of Tokyo, Tokyo, Japan; ^4^ Department of Radiation Oncology, Taipei Medical University Hospital, Taipei, Taiwan; ^5^ Department of Radiology, School of Medicine, College of Medicine, Taipei Medical University, Taipei, Taiwan; ^6^ Taipei Cancer Center, Taipei Medical University, Taipei, Taiwan; ^7^ TMU Research Center of Cancer Translational Medicine, Taipei Medical University, Taipei, Taiwan; ^8^ Graduate Institute of Biomedical Materials and Tissue Engineering, College of Biomedical Engineering, Taipei Medical University, Taipei, Taiwan; ^9^ International PhD Program in Biomedical Engineering, College of Biomedical Engineering, Taipei Medical University, Taipei, Taiwan; ^10^ Translational Laboratory, Research Department, Taipei Medical University Hospital, Taipei Medical University, Taipei, Taiwan

**Keywords:** head and neck cancer, chemotherapy resistance, chemotherapy, immunotherapy, combination therapy

## Abstract

Chemotherapy resistance is a huge barrier for head and neck cancer (HNC) patients and therefore requires close attention to understand its underlay mechanisms for effective strategies. In this review, we first summarize the molecular mechanisms of chemotherapy resistance that occur during the treatment with cisplatin, 5-fluorouracil, and docetaxel/paclitaxel, including DNA/RNA damage repair, drug efflux, apoptosis inhibition, and epidermal growth factor receptor/focal adhesion kinase/nuclear factor-κB activation. Next, we describe the potential approaches to combining conventional therapies with previous cancer treatments such as immunotherapy, which may improve the treatment outcomes and prolong the survival of HNC patients. Overall, by parsing the reported molecular mechanisms of chemotherapy resistance within HNC patient’s tumors, we can improve the prediction of chemotherapeutic responsiveness, and reveal new therapeutic targets for the future.

## Introduction

The global incidence of head and neck cancers (HNCs) continued to rise from 6.55 to 10.91% in the 10 years from 2008 to 2018. In 2018, over 1.9 million individuals were diagnosed with HNC including 354,864 lip and oral cavity, 177,422 larynx, 129,079 nasopharynx (NPC), 92,887 oropharynx, and 80,608 hypopharynx tumors (1). More than 90% of HNCs are squamous cell carcinoma (HNSCC) that occur from the mucosal epithelial tissue of oral cavity, oropharynx, and larynx (2). The common treatments include surgery, radiotherapy, chemotherapy, and concurrent chemoradiotherapy according to the stage of the disease, anatomical site, and surgical accessibility. Approximately 30~40% of stage I or II HNC patients are curable and show improved survival rates after surgery or radiotherapy alone. However, over 60% of stage III or IV HNC patients require advanced therapeutic options (3). For instance, chemotherapy or chemoradiotherapy is considered a promising approach to controlling tumor growth and prolonging survival rates in portions of stages III and IV HNC patients (4, 5). In a comparison of radiotherapy alone and with concurrent chemoradiotherapy, a meta-analysis of 19,248 HNC patients indicated that the additional use of chemotherapy with radiotherapy showed an increase in 5-year absolute survival (8.9% for oral cavity, 8.1% for oropharynx, 5.4% for larynx, and 4% for hypopharynx tumors) in a part of stages III and IV HNC patients (5, 6).

The standard chemotherapy regimens for stage III or IV patients are cisplatin, 5-fluorouracil (5-FU), and docetaxel/paclitaxel (7–9). A combined strategy of docetaxel, cisplatin, and 5-FU (TPF) treatment in a total of 358 unresectable HNSCC patients show significantly improved progression-free (11.0 month in TPF and 8.2 months in PF) and OS (18.8 months in TPF and 14.5 months in PF) (7). Another combined strategy of paclitaxel, cisplatin, and 5-FU (PPF) treatment on 80 stage III and IV HNSCC patients showed the 88% overall response rate and the 44% overall survival (OS) rates (10). Moreover, a 2016 phase III study report of PPF treatment in a total of 382 locally stage III and IV HNSCC patients indicated that a higher complete response (CR, 33% in PPF and 14% in PF) rate and a longer OS rate (43 month in PPF and 37 month in PF) (9). It is now widely accepted that TPF (docetaxel, cisplatin, and fluorouracil) treatment is the standard induction chemotherapy regimen (7, 11) and it has become the new standard for induction chemotherapy in the locally advanced HNSCC since the TAX323/EORTC24971 and TAX324 studies were published in Europe and the USA, respectively (7, 8). This TPF treatment is also used in the recurrent or metastatic HNSCC, which showed an improved overall response rate up to 44%, a median time to progression of 7.5 months, and a median OS of 11 months (12). TPF treatment may confer survival and organ preservation benefits in a part of HNSCC patients when it is administered safely by several clinical teams, though there is no consensus on the survival benefit (11).

However, the overall situation is still not optimal. The number of deaths from HNC continues to rise globally, from 586,400 deaths in 2008 to 980,787 deaths in 2018 (1). Chemotherapy resistance results in poor treatment outcome in HNC patients, and the reasons of chemotherapy resistance are multifaceted. Thus, building up a framework for understanding molecular mechanisms of chemotherapy resistance is the essential way to explore new therapeutic strategies (13). In this review, we summarize the molecular mechanisms of chemotherapy resistance after chemotherapies, such as cisplatin, 5-FU, and docetaxel/paclitaxel. We also describe up-to-date clinical trials, such as combination therapy and chemo-immunotherapy. Overall, this review provides intelligible and valuable information to readers to understand chemotherapy resistance in HNCs for effective treatment strategies.

## Chemotherapy Mechanisms

### Cisplatin

**Figure 1:**
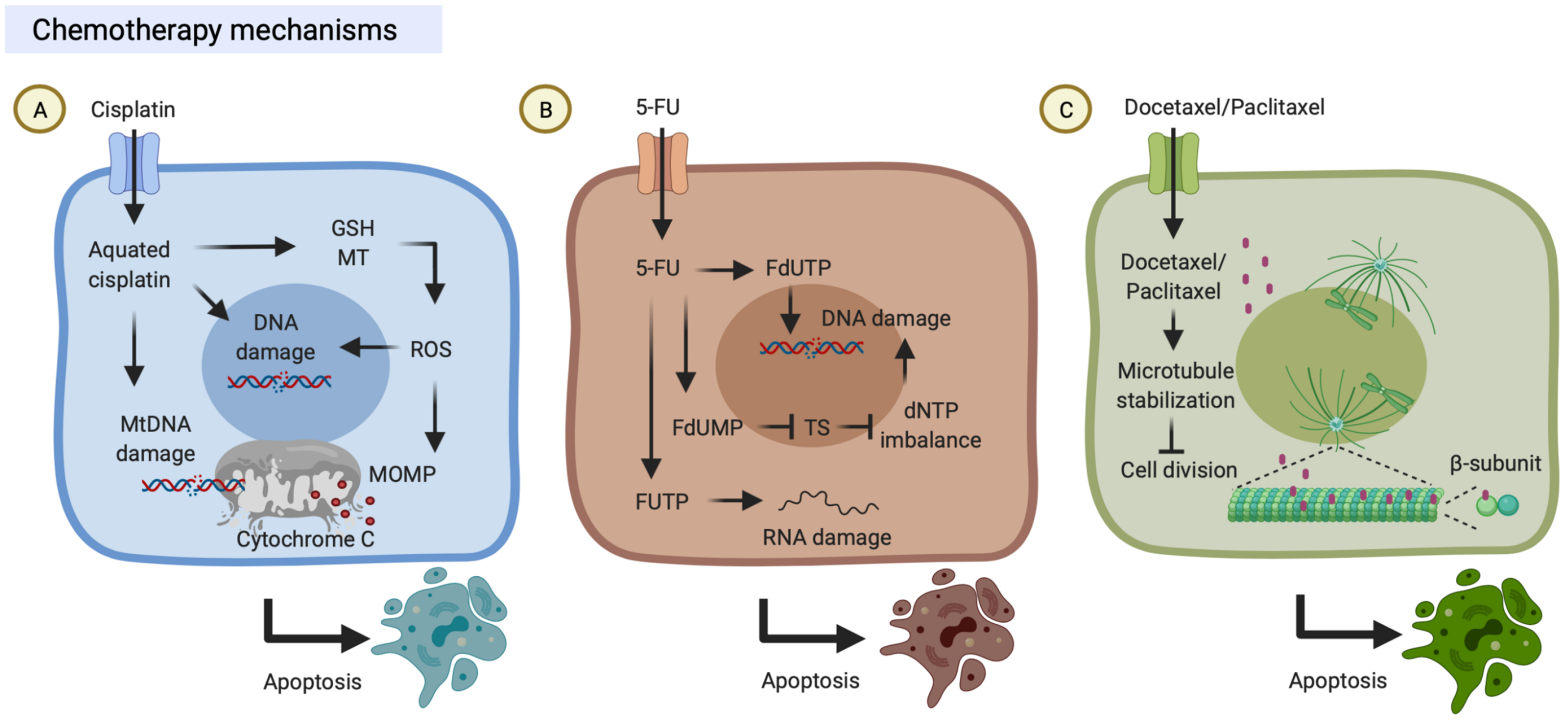
Chemotherapy mechanisms. **(A)** Cisplatin is transported into cells through copper transporters and is aquated in the cytosol. Aquated cisplatin induces DNA damage by binding to nuclear or mitochondrial DNA. On the other hand, aquated cisplatin also binds to cytoplasmic structures, including reduced glutathione (GSH) and metallothionein (MT), which results in the generation of reactive oxygen species (ROS) that trigger mitochondrial outer membrane permeabilization (MOMP). Both DNA damage and MOMP lead to cell death. **(B)** 5-Fluorouracil (5-FU) can be transported into cells by uracil transporters due to its uracil-like analog structure. Intracellular 5-FU is converted to three primary active metabolites which result in DNA/RNA damage: (i) fluorodeoxyuridine monophosphate (FdUMP) inhibits thymidylate synthase (TS); (ii) fluorodeoxyuridine triphosphate (FdUTP) is misincorporated into DNA; and (iii) fluorouridine triphosphate (FUTP) is incorporated into RNA. **(C)** Docetaxel/Paclitaxel can bind to microtubules which promotes tubulin assembly, suppresses microtubule dynamics and cell division, and ultimately results in cell death. These figures were created with BioRender.com.

The anti-tumor properties and contribution to clinics of the platinum-based drug, cisplatin, were discovered in the 1970s (14). Cisplatin is known to induce cytotoxicity to tumor cells through binding to DNA and impairing its repair mechanism. First, cisplatin can be transported into the cells through copper transporters and subsequently aquated due to low chloride concentrations in the cytosol (15). Aquated cisplatin induces DNA damage by binding to the guanine N^7^ position on either nuclear DNA or mitochondrial DNA. Finally, cisplatin-induced DNA damage leads to mitochondrial outer membrane permeabilization (MOMP). Bcl-2-associated X/Bcl interacting domain (BAX/BID) forms a pore to release mitochondrial protein cytochrome c into the cytoplasm. Released cytochrome c can activate the apoptotic protease-activating factor (Apaf)-1 apoptosome, which eventually results in the activation of caspases and induction of cell apoptosis. On the other hand, aquated cisplatin also binds to cytoplasmic molecules, including reduced glutathione (GSH) and metallothionein (MT), which results in the generation of reactive oxygen species (ROS) that also trigger MOMP and DNA damages (16) (**Figure 1A**).

### 5-FU

5-FU is an anti-metabolite drug and its anti-tumor properties were discovered in the 1950s, and it has been widely used to treat a range of cancers (17). 5-FU can be transported into the cells through similar transport mechanisms as uracil due to its uracil-like analog structure (18). Intracellular 5-FU is converted to three primary active metabolites: fluorodeoxyuridine monophosphate (FdUMP), fluorodeoxyuridine triphosphate (FdUTP), and fluorouridine triphosphate (FUTP). (i) FdUMP inhibits thymidylate synthase (TS) and results in dNTP imbalance which decreases levels of deoxythymidine triphosphate (dTTP) and increases levels of deoxyuridine triphosphate (dUTP) conversely (17, 19). (ii) FdUTP is misincorporated into DNA and increases the FdUTP/dTTP ratio, which results in DNA damage due to false nucleotide incorporation (17, 20). (iii) FUTP is incorporated into RNA, which results in disruption of RNA processing and function, including the processing of pre-ribosomal RNA, post-transcriptional modification of transfer RNA, and the splicing of pre-messenger RNA (17, 21–23) (**Figure 1B**).

### Paclitaxel/Docetaxel

Paclitaxel is both anti-cytoskeletal and anti-neoplastic drugs that were discovered in the 1990s, and it was subsequently approved for clinical use to treat several types of cancer (24). Paclitaxel binds to the β-subunit of microtubules (25), promotes tubulin assembly, and suppresses microtubule dynamics, which results in inhibition of mitotic blockage, chromosome segregation, and cell division (24). First, paclitaxel stabilizes microtubules and prevents them from disassembly; then chromosomes are unable to proceed to metaphase, and thus this mitotic blockage limits cell division and triggers cell apoptosis (24, 26, 27). It is worth noting that paclitaxel can also suppress microtubule detachment from centrosomes and reduces the tension on kinetochores that damages the bipolar attachment of sister chromatids and the interaction between kinetochores and spindle microtubules (28, 29) (**Figure 1C**). As an analog of paclitaxel, docetaxel is also an inhibitor of microtubule depolymerization mechanically (**Figure 1C**). Docetaxel and paclitaxel share most parts of their structures and mechanisms of action but differ in some aspects. Structurally, docetaxel (C_43_H_53_NO_14_) differs from paclitaxel (C_47_H_51_NO_14_) in the 10-position on the baccatin ring and in the 3’-position of the lateral chain. Efficiently, docetaxel is approximately twice higher binding affinity to tubulin compared with paclitaxel. Clinically, the respective response rates of paclitaxel and docetaxel were 43% in 28 HNC patients and 44% in HNC 38 patients, suggesting both paclitaxel and docetaxel are active in HNC patients (30).

## Chemotherapy Resistance

There are four main mechanisms that HNC cells acquire to avoid cell death following cisplatin (14, 16, 31, 32), 5-FU (33, 34), and paclitaxel/docetaxel treatments (35–37), including DNA/RNA damage repair, drug efflux, apoptosis inhibition, and epidermal growth factor receptor (EGFR)/focal adhesion kinase (FAK)/nuclear factor (NF)-κB activation. Below, we summarize these resistance mechanisms of chemotherapy regimens that occur in HNC cells and describe the most current reports available.

### Cisplatin

**Figure 2:**
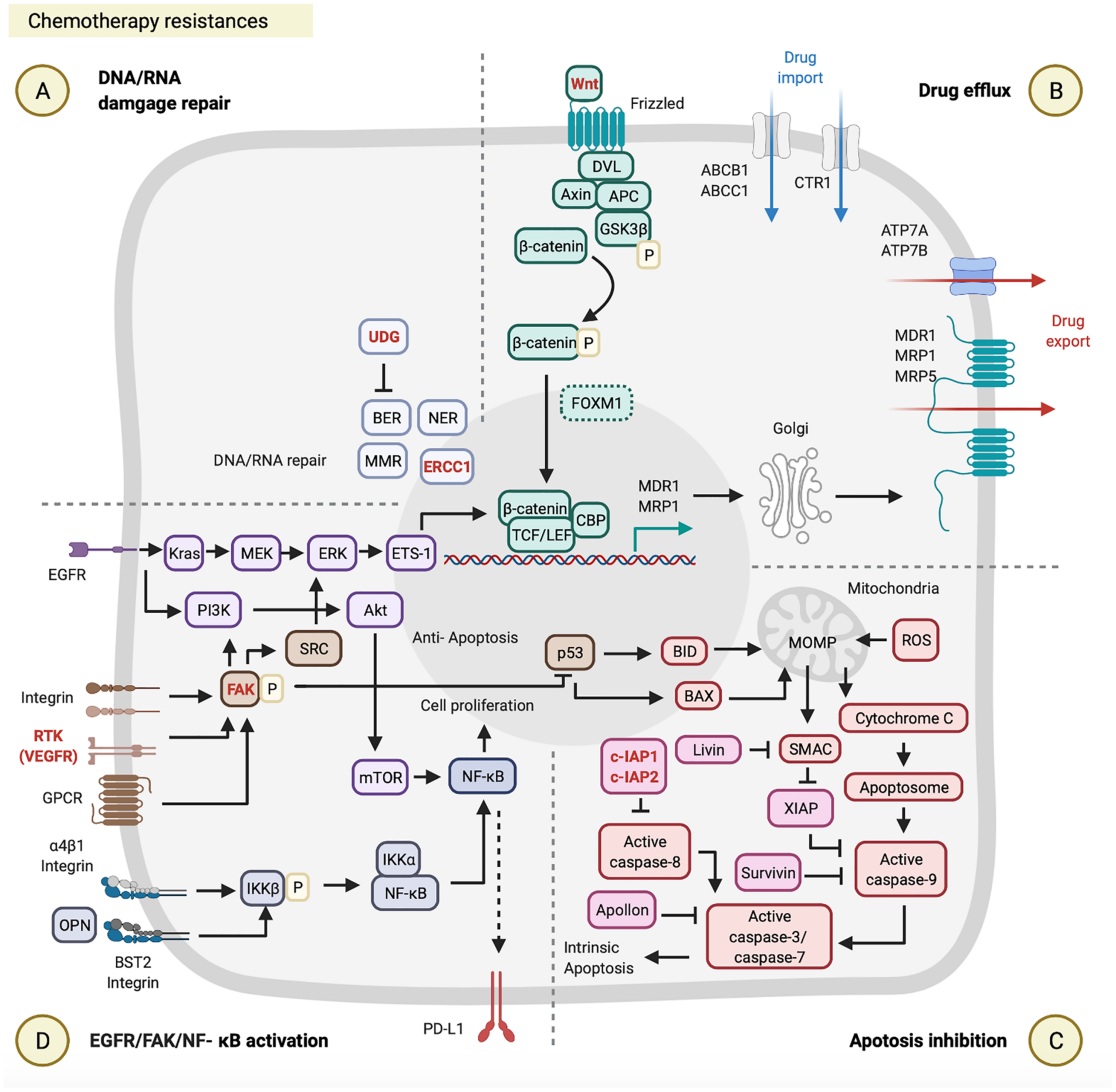
Chemotherapy resistances. Chemotherapy resistance is involved in **(A)** DNA/RNA damage, **(B)** drug efflux, **(C)** apoptosis inhibition, and **(D)** epidermal growth factor receptor (EGFR)/focal adhesion kinase (FAK)/nuclear factor (NF)-κB activation. These figures were created with BioRender.com.

DNA damage is repaired through four major mechanisms, including double-strand break repair (DSSR), mismatch repair (MMR), base excision repair (BER), and nucleotide excision repair (NER) (**Figure 2A**). NER is known as the primary strategy acquired by cancer cells to resist cisplatin-induced DNA damage. NER and its associated protein called the DNA excision repair protein ERCC1 were studied in several reports, and experimental evidence supports high EERC1 expression being associated with cisplatin resistance in HNSCC patients (38–40). For example, a previous study evaluated the effect of ERCC1 expression on the response to cisplatin in 57 patients with locally advanced unresectable HNSCC. Those HNSCC patients with high ERCC1 expression showed lower cisplatin treatment responses (50%, 13 out of 26 patients) and lower 2-year OS (44%), relative to those patients with low ERCC1 expression who showed higher responses (90.3%, 28 out of 31 patients) and better survival (74.2%) (39) (**Table 1**). Although a correlation between the ERCC1 expression level and cisplatin resistance was reported, the mechanism through which ERCC1 acts is not yet clarified and is still under investigation (**Figure 2A**).

**Table 1:** Markers of chemotherapy resistance in head and neck cancers (HNCs).

Drug	Type	Markers	Results	Ref.
Cisplatin	DNA repair	ERCC1	Thirteen of 26 HNSCC patients with high ERCC1 expression showed a lower cisplatin treatment response (50%) and lower 2-year OS (44%).	(39)
			In total, 1,263 HNSCC patients from 17 studies were performed in a meta-analysis. Pooled HRs with 95% confidence intervals (CIs) for OS and PFS were 2.14 and 2.60, respectively.	(40)
	Drug efflux	MDR1 MRP1	In total, 317 HNSCC patients with high expression level of MDR1 and MRP1 showed lower OS and PFS.	(41)
		β-catenin	Knockdown of β-catenin in HNSCC cell lines (SCC-15 and SCC-25) sensitized their response to cisplatin and reduced tumor growth.	(42)
	Apoptosis inhibition	c-IAP1	High cIAP-1 expression in 17 HNSCC patients was correlated with lymph node metastasis, an advanced disease stage, and poor prognosis.	(43)
		XIAP	High XIAP expression in 17 (20.83%) of 60 advanced HNSCC patients was associated with cisplatin resistance and poor outcomes.	(44)
		Apollon	In total, 80 HNSCC patients with high levels of Apollon expression also had shorter OS, compared to the group with low Apollon expression group.	(45)
		Livin	Livin-knockdown in HNSCC cell lines (SNU1041, PCI1 and PCI50) induced apoptosis and enhanced chemotherapy-induced apoptosis to cisplatin, 5-FU, and docetaxel.	(46)
	EGFR/FAK/ NF-κB activation	BST2	Knockdown of BST2 in HNC cell lines (HONE1, HNE1, and CNE2) sensitized their response to cisplatin and enhanced cisplatin-induced apoptosis. In total, high BST2 expression is associated with poor prognosis in 117 HNC (locally advanced nasopharyngeal carcinoma) patients with platinum-based chemotherapy	(47)
		KRAS	In total, 103 recurrent and/or metastatic HNSCC patients with the KRAS-variant had poor PFS when treated with cisplatin.	(48)
5-FU	Apoptosis inhibition	Livin	Livin-knockdown in HNSCC cell lines (SNU1041, PCI1, and PCI50) induced apoptosis and enhanced chemotherapy-induced apoptosis to cisplatin, 5-FU, and docetaxel.	(46)
		c-IAP2	Downregulation of cIAP2 enhanced the sensitivity of 5-FU-resistant HNSCC cell line (SAS) to 5-FU, with a significant increase in apoptosis.	(49)
Paclitaxel	Drug efflux	MDR1 MRP5	Depletion of MDR1 or MRP5 in paclitaxel-resistant HNSCC cell lines (CNE1, CNE2, and EC109) by siRNA blocked drug efflux, led to increased intracellular concentrations of paclitaxel and resulted in paclitaxel-induced cell death.	(50, 51)
	Apoptosis inhibition	Survivin	High survivin expression in HNSCC biopsies and HNSCC cell lines (Cal27, NT8e, CNE-2, 5-8F, and 6-10B) was associated with paclitaxel resistance and progression	(52–54)
Docetaxel	Drug efflux	MDR1	High MDR1 expression in HNSCC cell line (DR-Hep2) was associated with paclitaxel resistance	(55)
	Apoptosis inhibition	ROS	The docetaxel-resistant HNSCC cell line (DR-Hep2) increased the amount of mitochondrial DNA (mtDNA) and reduced the ROS generation.	(55)

OS, overall survival; HNSCC, head and neck squamous cell cancer; HR, hazard ratio; CI, confidence interval; PFS, progression-free survival; Ref, reference; 5-FU, 5-fluorouracil; ERCC1, excision repair cross-complementation group 1; MDR1, multidrug resistance 1; MRP1, multidrug resistance protein 1; cIAP2, cellular inhibitor of apoptosis protein 2; ROS, reactive oxygen species.

Drug efflux enables cancer cells to resist cisplatin by reducing intracellular drug levels. The ATP-binding cassette (ABC) transporter superfamily is one of the platinum-drug efflux transporters, which are mediated by multidrug resistance (MDR) genes (56). Some ABC transporter proteins were previously reported, including MDR1, MDR protein 1 (MRP1), MPR2, MPR3, and MPR5 (14, 56). Furthermore, the copper transporter family is another type of platinum-drug influx/efflux transporter, such as high-affinity copper uptake protein 1 (CTR1, involved in cisplatin influx) and two P-type ATPases (ATP7A and ATP7B, involved in cisplatin efflux) (15, 57, 58). Clinical data from The Cancer Genome Atlas database were used to evaluate the effects of expression of different types of platinum-drug efflux transporters on the response to cisplatin in 317 HNSCC patients. This evaluation indicated that HNSCC patients with cisplatin resistance and low survival were associated with the high MDR1 and MRP1 expressions by their tumor biopsy, but were not associated with ATP7B and MRP2 expressions (41), which highlights the critical roles of MDR1 and MRP1 in cisplatin resistance (**Figure 2B**; **Table 1**). It is worth noting that both MDR1 and MRP1 are regulated by the activation of the Wnt/glycogen synthase kinase (GSK)-3β/β-catenin pathway (42, 59, 60). The Wnt signaling pathway is initiated by the binding of the Wnt ligand to a Frizzled receptor. The GSK-3β protein complex [which includes axin, disheveled (DVL), adenomatous polyposis coli (APC), and GSK-3β] activates β-catenin by phosphorylation. Activated β-catenin further cooperates with the T-cell factor (TCF) transcription factor, lymphoid enhancer-binding factor (LEF), and CREB-binding protein (CBP) to initiate MRD1 and MRP1 expressions in the nucleus (61). Some very recent studies on HNCs provided evidence to support the correlation between the activation of the Wnt/GSK-3β/β-catenin pathway and cisplatin resistance (42, 62, 63) (**Figure 2B**; **Table 1**). For example, Long Li et al. investigated the effect of β-catenin on cisplatin resistance by using HNSCC cell lines (SCC-15 and SCC-25) and evaluated the cisplatin susceptibility of SCC-15/SCC-25 cells with β-catenin gene knockdown. After β-catenin gene knockdown in SCC-15/SCC-25 cells, low β-catenin expressing SCC-15/SCC-25 cells were inoculated into BALB/c nude mice. The tumor growth analysis showed that low expression of β-catenin in SCC15/SCC-25 cells could increase cisplatin sensitivity and reduce tumor progression after cisplatin treatment (42) (**Table 1**). Taken together, the expression level of β-catenin is associated with cisplatin resistance.

Apoptosis inhibition is one of the acquired capabilities used by cancer cells to resist cisplatin. The inhibitor of apoptosis protein (IAP) can prevent activation of the apoptosis signaling pathway by blocking caspases (64, 65). As to the apoptosis signaling pathway, cisplatin-induced DNA damages first causes p53 upregulation in the nucleus. Those upregulated p53 proteins bind to the upstream promoter region of BAX and BID to initiate their gene expressions (66). The BAX and BID apoptotic proteins permeabilize the outer mitochondrial membrane, which results in the release of cytochrome c and the second mitochondrion-derived activator of caspases (SMAC) into the cytoplasm. In the cytosol, cytochrome c further interacts with apoptotic protease activating factor 1 (APAF1) to form apoptosomes. These apoptosomes transactivate caspase-9, caspase-3, and caspase-7, and consequently leads to intrinsic apoptosis (67, 68) (**Figure 2C**). Regarding apoptosis inhibition by IAPs, as recently reported, high expressions of cellular inhibitor of apoptosis protein 1 (c-IAP1), X-linked inhibitor of apoptosis protein (XIAP), and Apollon observed in HNSCC patients were associated with the low survival rates and cisplatin resistance (43–45, 69) (**Figure 2C**; **Table 1**). For example, approximately 20.83% (17 out of 60) advanced HNSCC patients showed high XIAP expression by their biopsies, which were associated with cisplatin resistance (*p* = 0.036) and poor clinical outcomes (*p* = 0.025) (44) (**Table 1**). Another study of 80 HNSCC patients indicated that 60% (48 out of 80) of those patients exhibited high expressions of Apollon protein and mRNA, which was correlated to a low OS rate (median survival time: 28 months, *p* < 0.001) (**Table 1**).

EGFR/FAK/NF-κB are critical signal pathways activated and used by cancer cells to resist cisplatin. In the FAK pathway, FAK can be activated by receptor tyrosine kinases (RTKs), integrins, and G-protein-coupled receptors (GPCRs) (70). Activated FAK involves three major signaling pathways: SRC/extracellular signal-regulated kinase (ERK)/external transcribed spacer region (ETS)-1 (71, 72), phosphatidylinositol 3-kinase (PI3K)/Akt/NF-κB (47), and p53 (73, 74). FAK can (i) induce MDR1-mediated drug efflux through SRC/ERK/ETS-1/β-catenin pathway (70–72, 74, 75); (ii) promote cell proliferation through PI3K/Akt/mTOR/NF-κB pathway (70); (iii) also directly suppresses p53-caused apoptosis (73, 76) (**Figure 2D**). In the EGFR pathway, there are two primary pathways activated, such as KRAS/methyl ethyl ketone (MEK)/ERK and PI3K/AKT/mTOR in HNC cells (77, 78) (**Table 1**). The EGFR can (i) induce MDR1 and/or MRP-mediated drug efflux through KRAS/MEK/ERK/ETS-1/β-catenin pathway (48, 79, 80); (ii) can also promote cell proliferation through PI3K/Akt/mTOR/NF-κB pathway (**Figure 2D**). A preclinical study indicated that cisplatin-resistant HNSCC cell lines (HONE1, HNE1, and CNE2) highly express BST2, and BST2 can prevent cell apoptosis *via* the NF-κB pathway. Moreover, high BST2 expression levels can serve as an indicator of cisplatin resistance and poor prognosis in a total of 117 locally advanced NPC patients (47) (**Table 1**). This report provides a new aspect of the cisplatin resistance mechanisms, but the landscape of BST2/NF-κB pathway requires further investigation.

### 5-FU

DNA/RNA damage repair by MMR and BER is one of the acquired capabilities used by cancer cells to resist 5-FU. As far as we know, BER-mediated DNA repair can remove modified or inappropriate bases by uracil-DNA glycosylase (UDG), cleavage the phosphodiester bond at the resulting AP site by an endonuclease, clean-up the 3’ or 5’ terminal end, replace the excised nucleotides by a polymerase, and seal the final DNA nick by a ligase. In 5-FU resistant cancer cells, after FdUTP is incorporated into DNA, the UDG can lyse the uracil-deoxyribose glycosyl bond of the dUTP and 5-FdUTP residues in DNA (81). MER-mediated DNA repair corrects replication errors between base and base mismatches and the polymerase slippage products at nucleotide repeat sequences, such as insertion and deletion loops (81) (**Figure 2A**).

Apoptosis inhibitions by c-IAP2 and Livin were found in two HNSCC studies (46, 49). One study evaluated the role of cIAP2 based on DNA microarray data using parental and 5-FU-resistant HNSCC cell line (SAS). Overexpression of cIAP2 contributes to 5-FU resistance and a poor prognosis in those 5-FU-resistant SAS cells (49). Another study evaluated the role of Livin in the susceptibility of HNSCC cell lines (SNU1041, PCI1, and PCI50) to 5-FU. Unlike c-IAP2, Livin preferentially binds to SMAC and then prevents SMAC from blocking XIAP-mediated inhibition of caspase-9 (64, 65). *Livin* gene-knockdown in those three HNSCC cell lines (SNU1041, PCI1, and PCI50) enhanced 5-FU-induced apoptosis (46) (**Figure 2C**; **Table 1**).

### Paclitaxel/Docetaxel

Drug efflux plays a critical role in paclitaxel resistance. Several types of paclitaxel/docetaxel resistant HNC cells overexpress MDR1 and MRP5 (50, 51, 55), which rescues those cancer cells from paclitaxel/docetaxel induced cytotoxicity. Interestingly, both Hou et al. and Shi et al. pointed out that the axis of forkhead box protein M1 (FOXM1) and MDR1/MRP5 is a newly defined drug efflux mechanism in HNSCC cell lines (CNE1, CNE2, and EC109) (50, 51); however, the FOXM1/MPR5 or FOXM1/MDR1 axis has not yet been clarified in HNC cells. The potential molecular mechanism of FOXM1 was investigated in glioma stem cells. FOXM1, a downstream factor of the Wnt/β-catenin signaling pathway, supports β-catenin translocation to nuclei, combines TCF/LEF transcriptional factors, and thereby activates target genes (82) (**Figure 2B**), suggesting that FOXM1 may promote MRP5 expression through the Wnt/β-catenin signaling pathway.

Apoptosis inhibition is another strategy found in paclitaxel resistance. Liu et al. indicated that remodeling and spacing factor 1 (RSF1) inhibits cell apoptosis *via* promoting the NF-κB pathway. Activated NF-κB signaling triggers Survivin expression on HNSCC cell lines (CNE-2, 5-8F, and 6-10B) (52). Survivin can inhibit the active caspase-9, which blocks the apoptosis (53, 54, 83) (**Figure 2C**; **Table 1**), and also maintains the integrity of the mitotic spindle that suppresses aberrant mitosis from producing mitotic damage by paclitaxel (84). For example, approximately 72% (21 out of 29) HNSCC patients showed high survivin expression by in tumor biopsies, which were associated with p53 expression, paclitaxel resistance, and progression (53, 54). On the other hand, the docetaxel-resistant HNSCC cell line (DR-Hep2) increased the amount of mitochondrial DNA (mtDNA) and reduced the ROS generation. Although the mechanism remains unclear, Mizumachi et al. hypothesized that the mtDNA plays a critical role in docetaxel resistance through suppressing ROS generation from the mitochondrial respiratory chain (55).

Changes in microtubule assembly alter the sensitivity of cancer cells to paclitaxel due to mismatched binding of paclitaxel to β-tubulin isotypes. Common β-tubulin isotypes include βI, βII, βIII, βIVa, βIVb, βV, and βVI. In particular, it is widely accepted that increased levels of βIII-tubulin cause paclitaxel resistance by rendering microtubules less sensitive to its effects (85, 86). However, Nienstedt et al. found that over 90% of 445 HNSCC biopsies expressed TUBB3 (known as the βIII coding gene), with 69 of them (15.5%) with weak expression, 149 of them (33.5%) with moderate expression, and 188 of them (42.2%) with cancers. The TUBB3 expression level showed no significant correlation with clinical implications or treatment outcomes (87). Thus, the critical roles of β-tubulin isotypes in paclitaxel-resistant HNC patients need further investigation.

It is worth noting that multidrug cross-resistance mechanisms possibly occur on HNC cells. As previously reported, triple drugs (docetaxel, cisplatin, and 5−FU) −resistant HNSCC cell lines (Hep−2 and CAL−27) exhibited higher chemotherapy resistance, reduced apoptotic cell death, and an increased expression of MDR1, MRP2, ERCC1, CTR, Survivin, and TS (88), which suggests that multiple drug-resistant HNSCC cells can simultaneously have multidrug cross-resistance mechanisms, including DNA/RNA damage repair, drug efflux, and apoptosis inhibition.

## Chemotherapy Resistance in Head and Neck Cancer Stem Cells

A rare subset of cells with stem cell features known as cancer stem cells (CSCs) have been demonstrated to highly tumorigenic, metastatic, and therapeutic resistance in both chemotherapy and radiotherapy (89). In HNC biopsies, a subpopulation of cells identified as CSC with high expression of stemness-related markers CD44 and BMI-1 (90). The CSC-related carcinogenesis and therapeutic resistance require us to rethink how to re-evaluate the efficacy of cancer therapies with regard to CSC (91). The isolated sphere-forming CSC from primary HNCs exhibited stemness markers CK5, OCT4, SOX2, and Nestin. HNC CSCs showed chemotherapy resistance to cisplatin, 5-FU, paclitaxel, and docetaxel due to their increased expression levels of ABC transporters (92). Interestingly, the Wnt/β-catenin signaling contributes to abnormal ABC transporter-mediated drug efflux property in HNC CSCs. Moreover, the Wnt/β-catenin signaling also maintains the self-renew capacity and promotes the expression of stemness-associated genes SOX2, OCT4, CD44 in HNC CSCs (93). It is worth noting that targeting the Wnt/β-catenin signaling pathway in HNC CSCs has been reported as a promising strategy to reduce tumorigenicity, suppress drug efflux, induce cancer cell apoptosis, and increase sensitivity to chemotherapy (94).

## Strategies to Overcome Resistance and Improve Therapeutic Effects

### DNA/RNA Damage Repair

**Figure 3:**
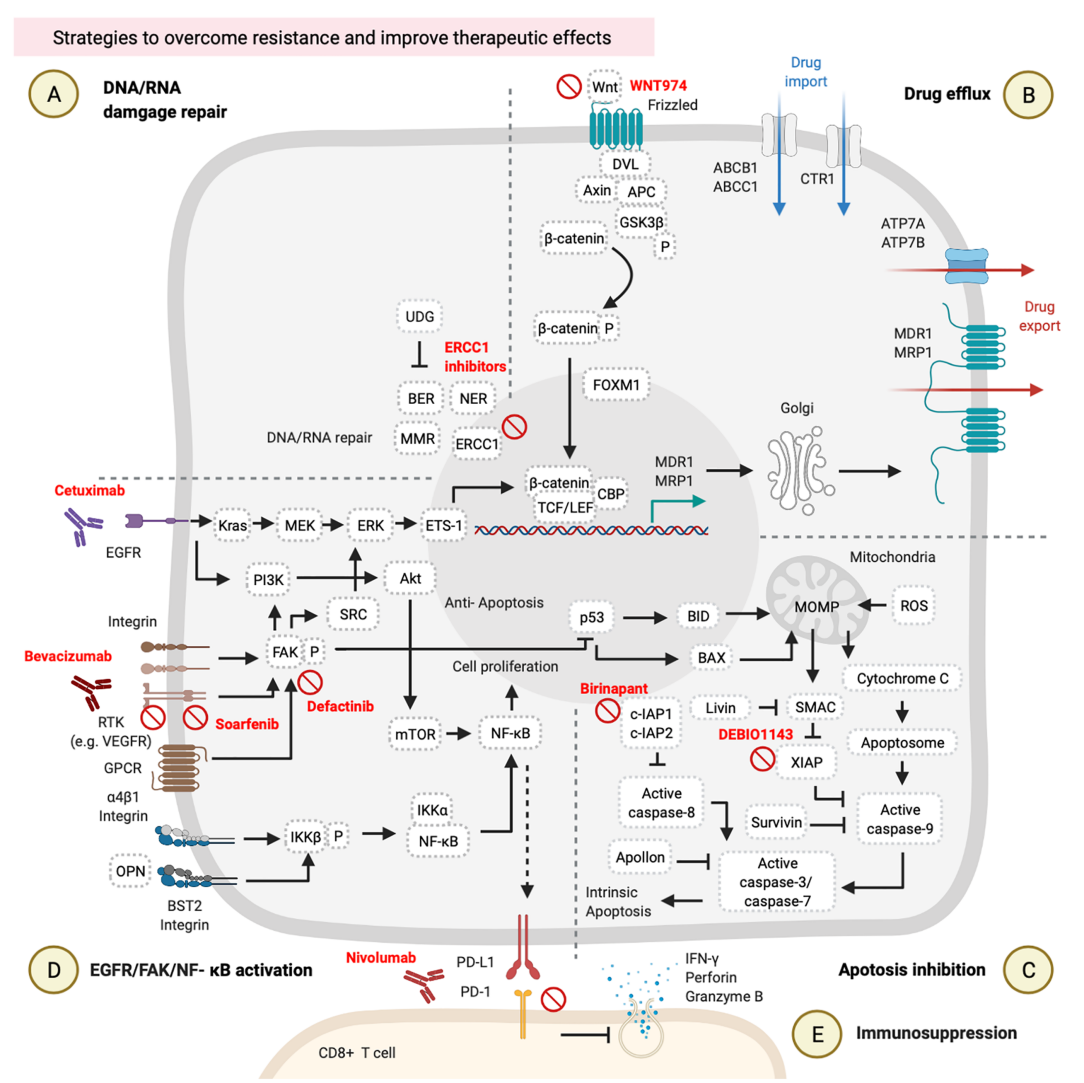
Strategies to overcome resistance and improve therapeutic effects. These involve **(A)** DNA/RNA damage, **(B)** drug efflux, **(C)** apoptosis inhibition, **(D)** epidermal growth factor receptor (EGFR)/focal adhesion kinase (FAK)/nuclear factor (NF)-κB activation, and **(E)** immunosuppression. These figures were created with BioRender.com.

DNA repair inhibitors can be used in combination with cisplatin. It is possible to inhibit different DNA repair pathways by blocking several targets, such as blocking ERCC1-XPF on the NER process by E-X PPI2 or E-X AS7 (95, 96). ERCC1–XPF is a structure-specific endonuclease that is required for repairing DNA damages caused by cisplatin. Two candidate inhibitors of ERCC1-XPF (E-X PPI2 and E-X AS7) reduced the ERCC1-XPF expression level, suppressed the NER process, and sensitized melanoma to cisplatin treatment (96) (**Figure 3A**). Although no reference describes the use of ERCC1-XPF inhibitors in HNCs so far, the previous references on other cancers suggest the possible use of DNA repair inhibitors in combination with cisplatin for HNCs.

### Drug Efflux

Wnt/β-catenin signaling inhibitors were examined in clinical trials. WNT974 was used as a Wnt/β-catenin inhibitor in a phase II trial of HNSCC patients (NCT02649530). These HNSCC patients received 10 mg of WNT974 daily for a month, and those patients showed tumor regression and improved disease-free survival (DFS) and OS rates with a tolerable toxicity profile. Combined treatment with cisplatin and WNT974 can be expected (97, 98) (**Figure 3B**).

### Apoptosis Inhibition

High expressions of IAPs by HNC cells can rescue them from cell apoptosis (64, 65). Targeting IAPs in cancer has become a new strategy to re-sensitize cancer cells to chemotherapies (99, 100). Birinapant is one of the IAP inhibitors in combination with carboplatin (a platinum analog of cisplatin) that can suppress cIAP1/2 expressions and improved the treatment outcomes of carboplatin in several different cancer cell lines, including ovarian cancer cell lines (S1-GODL, S8-GODL, S9-GODL, and Ovcar-3), lung cancer cell lines (A549, H226, and H460), cervical carcinoma cell lines (CaSki, HeLa, and SiHa), urinary bladder carcinoma cell line (5637, J82, and HT1197), colon cancer cell lines (DLD1, Colo205, and SW620), and HNSCC cell lines (PCI-1, PCI-9, PCI-13, PCI-52, and PCI-68) (101, 102) (**Figure 3C**, **Table 2**). Moreover, another IAP inhibitor, DEBIO1143 promotes apoptosis of cancer cells by mimicking the structure and activity of SMAC, which can block the XIAP and reactivate the caspase-9. A previous study on ovarian cancer cells suggests the possible use of DEBIO1143 combined with carboplatin to reverse carboplatin resistance and trigger cancer cell apoptosis (103). There is an ongoing phase II double-blind and randomized trial of combination treatment with DEBIO1143 and high-dose cisplatin chemoradiotherapy in high-risk locoregionally advanced HNSCC patients (104) (**Figure 3C**, **Table 2**).

**Table 2:** Summary of chemotherapy resistance in head and neck cancers (HNCs).

Drug	Effects	Resistance	Strategy
Cisplatin	Generate ROS Trigger MOMP Cause DNA/mtDNA damages	DNA repair	Unknown
		Drug efflux	Wnt/β-catenin inhibitor (WNT974)
		Apoptosis inhibition	IAP inhibitor (birinapant + carboplatin) (DEBIO1143 + cisplatin)
		EGFR/FAK/ NF-κB activation	EGFR inhibitor (cetuximab + cisplatin) VEGF inhibitor (sorafenib + cisplatin/5-FU) (bevacizumab + cisplatin/IMRT)
		Immunosuppression	PD-1 blockade (nivolumab + cisplatin) (pembrolizumab + platinum/5-FU)
5-FU	Generate FdUMP/FdUTP/FUTP FdUMP inhibits TS FdUTP causes DNA damage FUTP causes RNA damage	DNA repair	Unknown
		Drug efflux	Unknown
		Apoptosis inhibition	Unknown
		EGFR/FAK/ NF-κB activation	EGFR inhibitor (cetuximab + platinum/5-FU) VEGF inhibitor (sorafenib + cisplatin/5-FU)
		Immunosuppression	Unknown
Docetaxel/ Paclitaxel	Binds to microtubule β-subunit Promotes tubulin assembly Inhibits microtubule dynamics Limits cell division	DNA repair	Unknown
		Drug efflux	Unknown
		Apoptosis inhibition	Unknown
		EGFR/FAK/ NF-κB activation	VEGF inhibitor (bevacizumab + docetaxel/RT)
		Immunosuppression	Unknown

EGFR, epidermal growth factor receptor; ERCC1, excision repair cross-complementation group 1; IMRT, intensity‐modulated radiation therapy; MOMP, mitochondrial outer membrane permeabilization; mtDNA, mitochondrial DNA; ROS, reactive oxygen species; RT, radiation therapy; TS, thymidylate synthase; VEGF, vascular endothelial growth factor.

### EGFR/FAK/NF-κB Activation

Cetuximab is a monoclonal immunoglobulin G1 (IgG1) antibody that blocks the EGFR (105). The HNSCC patients with high EGFR expression are associated with chemotherapy resistance and poor treatment outcomes (48, 106). According to a phase III randomized trial of 117 recurrent/metastatic HNSCC patients, combined treatment with cetuximab and cisplatin improved the response rate from 10 to 26% and prolonged PFS from 2.7 to 4.2 months compared to cisplatin alone (107). Moreover, additional use of cetuximab with platinum/5-FU-based chemotherapy of 220 untreated recurrent or metastatic HNSCC patients significantly prolonged OS from 7.4 to 10.1 months in the group of patients who received chemotherapy plus cetuximab (108). Results of combined treatment with cetuximab and platinum/5-FU chemotherapy suggested that blocking the EGFR pathway by cetuximab is a potential way to improve the therapeutic effects of chemotherapy (**Figure 3D**, **Table 2**).

Vascular endothelial growth factor (VEGF) and its receptor (VEGFR) mediate tumor angiogenesis, which is associated with tumor progression and metastasis. Approximately 60~67% of NPC patients showed higher VEGF/VEGFR expressions and exhibited lower OS (109). The multi-kinase inhibitor, sorafenib, can block the autophosphorylation of several receptor tyrosine kinases (RTKs) such as the VEGFR and platelet-derived growth factor receptor (PDGFR) (110). In a phase II study, the combination of sorafenib, cisplatin, and 5-FU improved the objective response rate to 77.8% and prolonged the progression-free survival to 7.2 months in 54 recurrent or metastatic NPC patients (111) (**Figure 3D**, **Table 2**).

Bevacizumab is an antibody against the VEGF approved by the US Food and Drug Administration (FDA) for combined treatment with chemotherapy of lung cancer in 2006, recurrent ovarian cancer in 2016, and non-squamous non-small cell lung cancer in 2018 (112). So far, bevacizumab has been combined with chemotherapy in several clinical trials of other types of cancers, including HNCs (**Figure 3D**, **Table 2**). In a phase II study, combined treatment with intensity‐modulated radiation therapy (IMRT), cisplatin, and bevacizumab on 42 previously untreated stages III and IV advanced HNSCC patients improved their 2-year PFS rate to 75.9% (113). In another phase II study, combined treatment with radiotherapy, docetaxel, and bevacizumab on 30 previously untreated locally advanced HNSCC patients prolonged their 3-year PFS rate to 61.7% (114) (**Figure 3D**, **Table 2**).

Defactinib (also known as VS-6063) is an inhibitor of FAK (115), a primary downstream signal transducer of the VEGFR (116) (**Figure 3D**, **Table 2**). There are no clinical trials using defactinib on HNC patients, but there are on other types of cancers. Combined treatment with docetaxel and defactinib reduced the cell viability of docetaxel-resistant prostate cancer cells, suggesting that a combination of defactinib and docetaxel represents a strategy to overcome docetaxel-resistant prostate cancer (117). However, additional use of defactinib on 344 malignant pleural mesothelioma (MPM) patients who received first-line chemotherapy did not improve PFS, suggesting that the use of defactinib with chemotherapy requires further consideration (118) (**Figure 3D**, **Table 2**).

### Immunosuppression

Treatment of HNC cells with cisplatin or 5-FU upregulates the expression of programmed cell death ligand 1 (PD-L1) *via* the NF-κB pathway. PD-L1-expressing cancer cells can suppress cytotoxic activity and proliferation of CD8^+^ T cells by a PD-L1/programmed cell death (PD)-1 interaction (119–121). A preclinical trial revealed that the concurrent use of cisplatin and either an anti-PD-1 or anti-PD-L1 antibody suppressed tumor growth and prolonged survival in the HNC mouse model. This combined treatment did not produce side effects of decreasing the function of and the number of immune cells or increasing cisplatin-induced toxicities (121) (**Figure 3E**, **Table 2**). On the other hand, a clinical trial of recurrent or metastatic HNSCC patients revealed that nivolumab (an anti-PD-1 antibody approved by the US FDA) improved OS from 5.1 to 7.5 months, and 6-month PFS from 9.9 to 19.7%, and the response rate from 5.8 to 13.3% in these patients who had been pretreated with cisplatin (122). Two years of a follow-up study of the same trial revealed that these recurrent or metastatic HNSCC patients who had received both nivolumab and cisplatin showed higher 24-month OS (16.9%) compared to patients who received only cisplatin (6.0%) (123) (**Figure 3E**, **Table 2**). Another clinical trial of recurrent or metastatic HNSCC patients showed that pembrolizumab combined with platinum and 5­FU is an appropriate first line treatment that improved OS up to 13.0 months (124). Thus, the concurrent treatment of immune checkpoint inhibitors and chemotherapy could be considered a promising strategy for PDL1 highly expressed HNC patients.

### Limitations and Moving Forward

Unfortunately, the combined treatment approaches with chemotherapy and an inhibitor of DNA/RNA damage repair (such as an ERCC1 inhibitor, ERCC1-XPF) or drug efflux (such as a Wnt/β-catenin inhibitor, WNT974) are still under investigation, and none of them had clinical trials for HNSCC patients in the past (96, 98). Currently, there are two types of combined treatment with IAP inhibitor with platinum-based chemotherapy (birinapant and carboplatin; DEBIO1143 and cisplatin) reported (102, 104). Owing to high cIAP1 and XIAP expressions on cisplatin-resistant HNSCC patients, blocking cIAP1 or XIAP could be a possible strategy to improve cisplatin treatments. The birinapant inhibits cIAP1/2 to reactivate caspase-8, and the DEBIO1143 suppresses XIAP to reactivate caspase-9 both facilitates cancer cell apoptosis, which results in synergistic anti-tumor effects in combination with cisplatin (or carboplatin). On the other hand, due to high EGFR and VEGFR expressions on HNSCC patients with chemotherapy resistance and poor prognosis, there are multiple combined treatment trials with EGF/EGFR or VEGF/VEGFR axis inhibitor and chemotherapy reported, including cetuximab and cisplatin (107); cetuximab and platinum/5-FU (108); sorafenib and ciplatin/5-FU (111); bevacizumab and cisplatin/IMRT (113); bevacizumab and docetaxel/RT (114). Blocking EGF/EGFR or VEGF/VEGFR axis can suppress downstream FAK and PI3K signal pathways, which hampers MDR1-mediated drug efflux and p53-caused cell apoptosis. Blocking EGF/EGFR or VEGF/VEGFR axis could be the reason why these combinations increased the chemotherapy treatment’s effectiveness; however, these combinations’ mechanisms should be clarified in more detail and demonstrated the preclinical/*in vitro* findings in clinical aspects. Moreover, it is concerned that the expression level of EGFR and VEGF/VEGFR could impact the therapeutic effects of these combinations because only part of HNSCC patients showed high EGFR and VEGF/VEGFR expressions (109). Thus, an evaluation for these expression levels before considering combination treatment is required. Furthermore, it is worth noting that immune checkpoint inhibitors plus chemotherapy have become a promising strategy. Chemotherapeutic agents induce immunogenic cell death (ICD), which facilitates antigen cross-presentation and cytotoxic T cell generation. Immune checkpoint inhibitors can further rescue cytotoxic T cells from cancer cells and reinvigorate cytotoxic T cell function. The beneficial combination between chemotherapy and immune checkpoint inhibitors has recently been demonstrated as a first-line treatment of recurrent HNSCC patients (124).

## Conclusions

Chemotherapy resistance largely influences the therapeutic efficacy and results in poor prognoses in HNC patients. This review summarizes and updates the mechanisms underlying chemotherapy resistance on HNCs. Four primary resistance mechanisms, including DNA/RNA damage repair, drug efflux, apoptosis inhibition, and EGFR/FAK/NF-κB activation after cisplatin, 5-FU, and paclitaxel/docetaxel treatments, have been described. The corresponding strategies to those four mechanisms are listed, which can be translated into developing innovative cancer therapeutics to overcome chemotherapy resistance in HNC patients.

## Author Contributions

YK, C-YC, H-LL, J-FC, and Y-JC were involved in the design, wrote the article, and supervised the research. All authors contributed to the article and approved the submitted version.

## Conflict of Interest

The authors declare that the research was conducted in the absence of any commercial or financial relationships that could be construed as a potential conflict of interest.

## Abbreviations

5-FU, 5-fluorouracil; APC, adenomatous polyposis coli; BAX, Bcl-2-associated X; BID, Bcl-interacting domain; BER, base excision repair; BIRC1, baculovirus inhibitor of apoptosis protein repeat (BIR)-containing protein 1; CBP, CREB-binding protein; cIAP, cellular inhibitor of apoptosis protein; CR, complete response; CTR1, copper uptake protein 1; DSSR, double-strand break repair; dTTP, deoxythymidine triphosphate; dUTP, deoxyuridine triphosphate; DVL, disheveled; ERCC1, excision repair cross-complementation group 1; FAK, focal adhesion kinase; FdUMP, fluorodeoxyuridine monophosphate; FdUTP, fluorodeoxyuridine triphosphate; FUTP, fluorouridine triphosphate; GPCR, G-protein-coupled receptor; GSH, reduced glutathione; HNC, head and neck cancer; HNSCC, head and neck squamous cell carcinoma; IAP, inhibitor of apoptosis; MDR, multidrug resistance; MDR1, multidrug resistance 1; MMR, mismatch repair; MOMP, mitochondrial outer membrane permeabilization; MPM, malignant pleural mesothelioma; MRP1, multidrug resistance protein 1; MRP3, multidrug resistance protein 3; MRP5, multidrug resistance protein 5; MT, metallothionein; NF-κB, nuclear factor-kappa B; OS, overall survival; PDGFR, platelet-derived growth factor receptor; PFS, progression-free survival; ROS, reactive oxygen species; RSF1, remodeling and spacing factor 1; SMAC, second mitochondria-derived activator of caspases; TCF, T-cell factor; TS, thymidylate synthase. UDG, uracil-DNA glycosylase; VEGF, vascular endothelial growth factor.
